# Shikonin Production by Callus Culture of *Onosma bulbotrichom* as Active Pharmaceutical Ingredient

**Published:** 2018

**Authors:** Fereshteh Bagheri, Reza Tahvilian, Naser Karimi, Maryam Chalabi, Mahsa Azami

**Affiliations:** a *Pharmaceutical Sciences Research Center, School of Pharmacy, Kermanshah University of Medical Sciences, Kermanshah, Iran. *; b *Department of Biology, Faculty of Sciences, Razi University, Kermanshah, Iran. *; c *Department of Immunology, School of Medicine, Medical Biology Research Center, Kermanshah University of Medical Sciences, Iran.*

**Keywords:** *Onosma bulbotrichom*, Germination, Callus, Indole-3-acetic acid, 2, 4-Dichlorophenoxyacetic acid, HPLC

## Abstract

The objective of this research was *in-vitro* germination and callus induction of *Onosma bulbotrichum* (*O.* *bulbotrichum*) as a medicinal herb which belongs to Boraginaceae family. For germination, the seeds were cultured on growth regulator-free MS medium and for callus induction, seeds were sown on modified MS medium containing different concentrations of kinetin (kn)- Indole-3-acetic acid (IAA) and kn- 2,4-D (2,4-dichlorophenoxyacetic acid), respectively. The plates were maintained in the dark at growth chamber. After 7 days seed germination on hormone-free medium and after 10 days callus initiation on modified medium in the presence of hormones was occurred. The maximum pigmented callus (100%) was observed on modified MS medium with a combination of 0.2 mg.L^-1^ IAA + 2.10 mg.L^-1^ kn. Shikonin determination was performed by HPLC method. In addition, total hydroxynaphtoquinons as polyphenols in sum of callus and culture medium were measured by spectrophotometric method and revealed that total naphtoquinones content at IAA was more than 2, 4-D.

## Introduction

Boraginaceae family members such as *Onosma*, *Lithospermum*, *Arnebia* and *Alkanna* are well known as medicinal plants which produced many valuable secondary metabolites such as hydroxynaphthoquinone pigments. *Onosma* species are spread in Mediterranean region, central and western Asia, growing in dry, sunny, rocky, sandy and steppe habitats (1, 2). The bioactive constituents which are existed in *Onosma,* consist of rosmarinic acid and hydroxynaphthoquinone derivatives, chiefly shikonin and alkanin. Traditionally, these plants are used for treatment of a wide range of disorders such as wound healing, tumors, bronchitis, tonsillitis and hemorrhoids (3, 4). Scientific researches proved that naphtoquinone derivatives especially shikonin show apoptosis inducing and antireplicative activity against human immune- deficiency virus (HIV), also antitumor activity against human breast cancer and *etc.* (5-10). Therefore, the biotechnological approaches such as cell/tissue culture have been applied in order to produce these pharmaceutical substances resembling those accumulated in the root bark of the original plants. Over the last decades, many research projects have been done *in-vitro* production of shikonin and its derivatives by cell/tissue culture techniques. For example, callus induction of *Lithospermum erythrorhizon* (11), callus culture of *Echium lycopsis* (13), callus induction of *Arnebia euchroma *(14), shoots culture, suspension culture and protoplast culture of *Lithospermum erythrorhizon* (3, 6 and 15), callus culture of *Echium italicum* L. (16), as well as callus and cell suspension culture of *Arnebia euchroma* (17, 18). *Onosma bulbotrichum* is a member of Boraginaceae that is well- known as a rich source of secondary metabolites. The roots of this medicinal plant traditionally used for burn and wound-healing as ointment for thousands of years (19). With regards to rare geographical growing places and farming problems of *Onosma bulbutrichum*, economic and large scale production of shikonin is almost impossible. Therefore, the aim of this study was to utilize a certain method for germination and tissue culturing of *O. bulbotrichum *seeds. 

## Experimental

*Reagents and materials*

*Onosma bulbotrichum *(*O. bolbutrichum*) was gathered from Harsin (1530 m, 35° 3′ 0″N, 46° 16′ 12″E) in July 2013, Kermanshah province, Iran. The taxonomic identification of plant materials was confirmed by a senior plant taxonomist, Dr. N. Jalilian, at Kermanshah Agriculture and Natural Resource Research Center, Iran. Shikonin was purchased from Sigma-Aldrich. HPLC grade methanol was obtained from Merck (Darmstadt, Germany). Chloroform and acetone were purchased from Merck. 

*Seed sterilization*

The seeds of *O. bolbutrichum* were thoroughly washed in a running tap water for 20 min. These seeds were then surface- disinfected by maintaining in 55 °C water bath for 10 min and immediately transferred to an ice bath. Next step of sterilization performed by 70% (v/v) ethanol for 30 sec and washing 3 times with distilled water to remove the ethanol, followed by 0.5% (v/v) sodium hypochlorite for 10 min and rinsed three times with sterile distilled water. Subsequently, to remove the hard and thick shell of seeds, equal contents of surface sterilized seeds were separated and carefully soaked in 15% H_2_O_2_ for 5 min and washed four to five times with sterile distillate water again (20). The thick cortex of seeds was gently removed using sterile scalpel. 

*Germination and callus initiation*

At all stages, germination and callus culture media based on MS and 1/2 MS basal medium with slightly changes containing replacement of appropriate amount of nitrate with ammonium and 5% (w/v) sucrose (50 g.L^-1^) was used. The pH was adjusted at 5.6-5.7 for seed germination and 5.8 for initiation and maintenance of callus.

Eight sterilized seeds were cultured in each petri dish containing hormone-free MS and MS medium (Murashige and Skoog) with 8% agar supplemented with 2.10 mg.L^-1^ kinetin as a cytokine phytohormone and auxins: 2, 4-D (0.2, 0.5 and 1 mg.L^-1^) and IAA (0.2, 0.5 and 1 mg.L^-1^). The plates were placed in a growth chamber at 23 ± 2 °C under dark condition. Growing plantlets were appeared in hormone-free MS medium and calli in MS medium in combination with hormones after 7 and 10 days respectively. Plantlets and calli were sub cultured with 2 week intervals in 1/2 MS solid medium composed of 2.10 mg.L^-1^ kn + 0.2 mg.L^-1 ^2, 4-D and 2.10 mg.L^-1^ kn + IAA. Then, 500 mg of one month-old undifferentiated callus were transferred into liquid medium in a 250 mL erlenmeyer flask containing 100 mL 1/2MS medium supplied with different concentrations of 2, 4-D + 2.10 mg.L^-1^ kn and IAA (0.2 mg.L^-1 ^+ 2.10 mg.L^-1^ kn). All cultures were maintained at 23 ± 2 °C on a rotary shaker with 100 rpm in darkness. The calli were sub-cultivated routinely every 2 weeks. Shikonin production induced in the callus cultures on 1/2MS medium. All of these stages were served under sterile condition.

*Statistical analyses*

For calculation of callus induction rate, the number of callus in each individual treatment was recorded by counting and converting to a percentage. Six replicates and eight seeds in each replicate were applied. Analysis of variance (ANOVA) was used to evaluate the results; *p* < 0.05 and *p* < 0.01 were considered significant. Data were analyzed using Statistica software. All results are shown as means ± SE.

*Extraction*

Red pigments of two and four-week-old fresh callus tissues cultured at liquid medium were completely extracted with acetone. The obtained extract was filtered and dried at room temperature to yield shikonin derivatives extract, which can be stored at 4 °C and used for further studies. Shikonin in suspension culture medium was extracted again with 150 mL of chloroform for 24 h at room temperature in the dark. Subsequently, content of total polyphenols and shikonin were determined by spectrophotometric and HPLC apparatus, respectively. The total shikonin yield reported by HPLC method that originated from 1/2MS medium supplemented by IAA 0.2 mg.L^-1^ and kinetin is the sum of intracellular and extracellular shikonin yields.

*Total phenolics determination method*

*Gallic acid curve standard*

The method described by Gao *et al.* (21) was applied for total phenolic compounds measurement of sample extracts containing callus, liquid culture medium and nature root of plant, in which gallic acid as standard and folin-ciocalteu’s reagent were utilized. A stock solution of gallic acid (100 μg/mL) using 50% methanol was prepared and diluted with double distilled water. Various concentrations of gallic acid (10, 20, 30, 40 and 50 mg.L^-1^) was taken and mixed by 5 mL of 10% folin solution. After 3 to 8 min incubation and under dark condition, 4 μL of 7.5% sodium carbonate solution was added to the mentioned solution. The absorbance of considered concentrations of gallic acid solution was measured at 765 nm using UV-VIS spectrophotometer Shimadzu UV mini-1240. The blank sample was prepared in the same way with 50% methanol instead of gallic acid. Ultimately, the standard curve was plotted using obtained absorbance of gallic acid.

*Total phenolic compounds in extracts*

One- hundered μL of each plant extract was mixed by 500 μL of 10% folin solution. Above mentioned procedure was applied to recognize total polyphenols presence in samples but gallic acid was replaced by different concentrations of extract. The absorbance of all prepared samples was measured at 765 nm using a UV-VIS spectrophotometer. Finally, total polyphone of each sample based on gallic acid standard curve was calculated. All results were expressed as μg.mg^-1^ gallic acid.

*HPLC–DAD Chromatographic method*

Quantitation of shikonin was achieved according to previous report (22). Analysis was performed using an Agilent-1100 HPLC instrument equipped with a Waters Symmetry Shield column (C18, 4.6 mm × 150 mm); with the volume injection set to 20 μL and a DAD (G1315C) coupled with a software HPLC/DAD Chem Station (Rev.A.06.03) was used at a flow rate of 1.5 mL.min^-1^. The analysis was performed at room temperature. The mobile phase consisted of a mixture of 85% of A (MeOH) and 15% of B (water) during 20 min. Stock solution of shikonin (0.5 mg.mL^-1^) was prepared in methanol solution.

**Table 1:** Pigmented and non-pigmented callus percentages in presence of different concentrations of auxin hormones. Mean comparisons were performed by Tukey’s multiple range tests

	Non pigmented callus (%) mean ± SE	Pigmented callus (%) mean ± SE
IAA (mg.L-^1^)			
0.2	There were no non pigmented callus	68.33 ± 0.28	
0.5		56.67 ± 0.17	
1		36.67 ± 0.24	
2, 4-D (mg.L-^1^)			
0.2	41.66 ± 0.38	30 ± 0.54	
0.5	61.66 ± 0.37	15 ± 0.33	
1	86.66 ± 0.24	8.33 ± 0.33	

^*^Represents significant difference (*p* < 0.05).

**Table 2:** The fresh weight percentages of calli. The values were means of six replications ± standard error had significant differences at *p* < 0.01.

	Free hormone	IAA	2,4-D
Fresh weight	199.25 ± 2.8^٭٭٭^	120.8 ± 3.16^٭٭٭^	429.1667 ± 3.53

^*^Represents significant difference at *p* < 0.01 (^***^).

**Figure 1:**
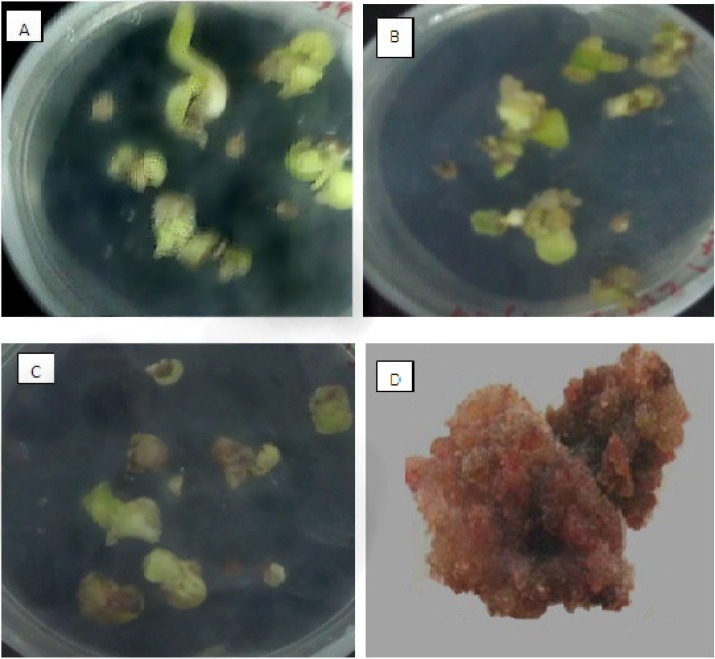
Seed germination and callus induction of *O. bolbutrichum*. (A) germination of seeds on free growth factor MS medium (B) appearance of callus without pigment production on MS medium with 2.10 mg.L^-1^ kn and 1 mg.L^-1^ 2, 4-D. (C) pigmented and non-pigmented calli on MS medium supplement with 2.10 mg.L^-1^ kn and 0.2 mg.L^-1^ 2, 4-D. (D) dye formation in liquid 1/2MS medium containing 0.2 mg.L^-1^ IAA

**Figure 2.**
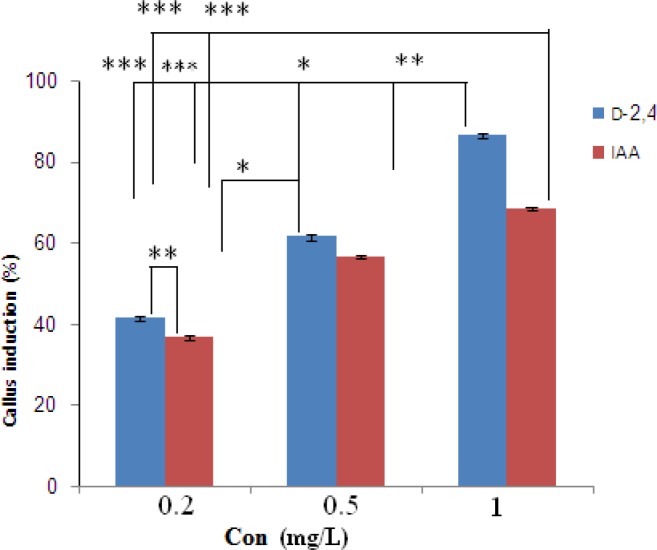
Effects of different concentrations of growth factors on callus initiation of *O. bolbutrichum *seed culture. *Represents significant difference at *p* < 0.05 (*) and *p* < 0.01(**, ***).

**Figure 3:**
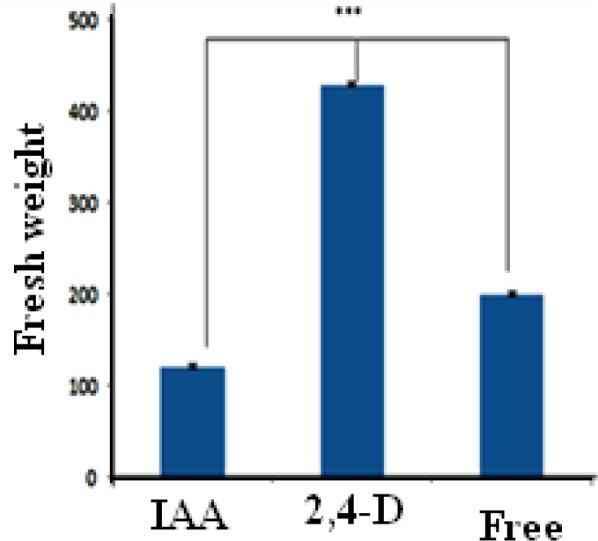
Effects of auxin kinds on growth of *O. bolbutrichum* calli cultured on MS liquid medium, significant at *p* < 0.01

**Figure 4:**
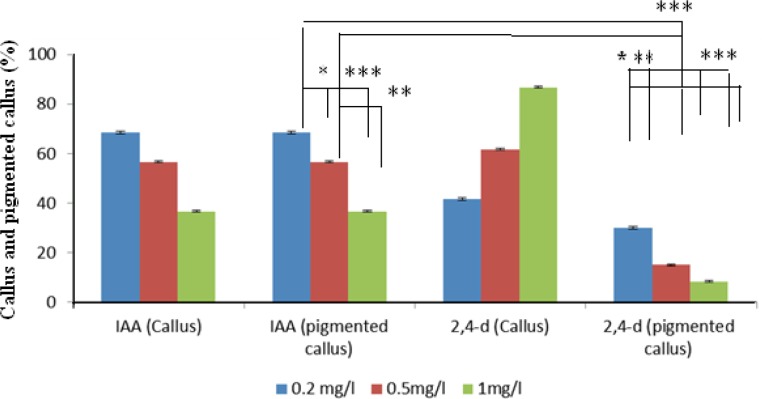
Effects of auxin types on red pigmented and nonpigmented callus production in callus cultured on modified MS medium at 25 °C in the dark. Error bar: SE. (n = 6). *Represents significant difference at *p* < 0.05 (*) and *p* < 0.01 (**, ***).

**Figure 5:**
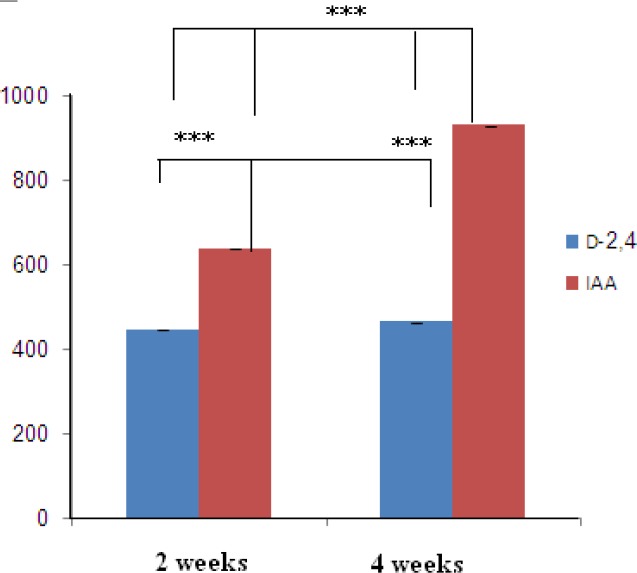
Total phenolics of *O. bolbutrichum*. There were significant differences (*p* < 0.01) among total phenolics of the callus and its medium extract supplemented by IAA. While the level of phenolic compounds in sum of callus and its medium extract treated 2, 4-D were not significantly different

**Figure 6:**
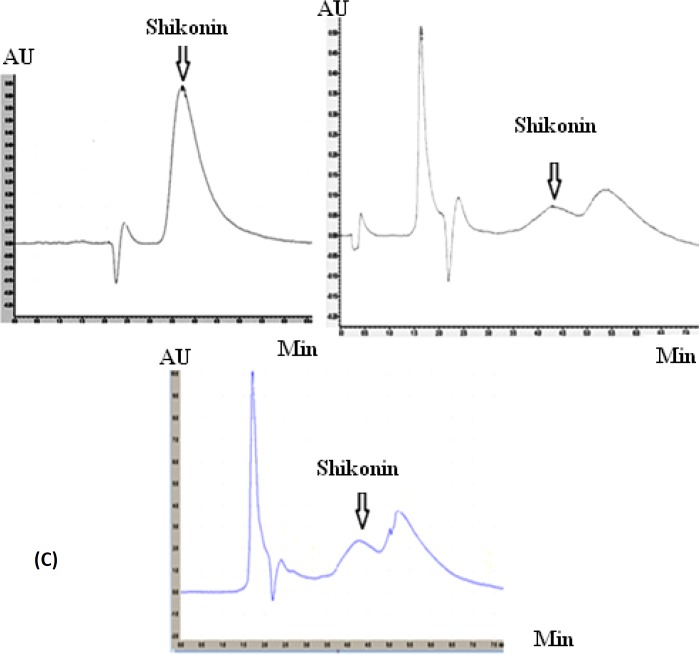
(A) Chromatogram of HPLC analysis belong to shikonin standard in 20 µg.mL^-1^, (B) extract of natural root bark and (C) callus towards its medium containing 0.2 mg.L^-1 ^IAA in 5 replicates

**Figure 7.**
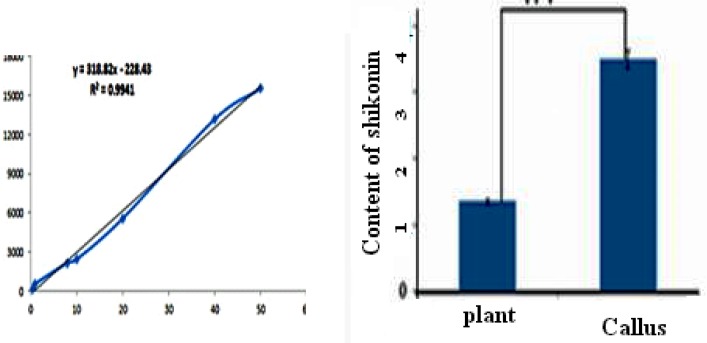
Shikonin accumulation in sum of callus and its medium containing IAA 0.2 mg.L^-1^ after 4 week culture and natural root extracts. Error bar: SE, (n = 5

*Standard curve of shikonin*

For plotting the standard curve, different concentrations of shikonin was prepared by methanol (0.5-40 ug.mL^-1^). Twenty uL of shikonin dilutions were injected to HPLC and monitored at 520 nm. The area of the related peaks was calculated and the standard curve was plotted.

## Results and Discussion

*Callus culture*

Since the seeds were collected from nature, they had high bacterial and fungal contaminations. The best method for surface sterilization was using ethanol, sodium hypochlorite and H_2_O_2_ after water bath treatment. Immersing the seeds in 55 °C water may cause starting fungal spore growth. Growing spores are halted immediately after putting them on the ice. Ethanol could remove the bacteria from surface of the seed. Also Hypochlorite and H_2_O_2_ can destroy the fungal and bacteria on the surface of them. 

Cultured seeds of *O. bolbutrichum *in free hormone MS media showed a high capacity for germination without any significant differences (more than 95%) while both callus initiation and maintenance of plant cell culture depend on media supplemented by auxin and cytokinin growth factors (Figure 1). As shown in the Figure 1, obtained callus of cultured seeds in terms of color production developed into two types of callus with different colors, including non-pigmented callus and red pigmented callus. Callus cultures that originally derived from *O. Bolbutrichum *seeds could be initiated in MS medium supplied with 2.10 mg.L^-1^ kinetin and different auxins. As illustrated in Figure 2, the highest percentage of callus induction was observed in 2, 4-D 1 mg.L^-1^. But these calli failed to produce naphthoquinone pigments (Table 1). Statistical analysis showed that the callus induction rate varied significantly between 2 different types of auxins used.

The effects of two types of auxins applied in MS media on the pigmented callus induction rate are displayed in Figure 2, in which callus induction rate occurred in the presence of 0.2 mg.L^-1^ IAA. Moreover, there were important differences between different auxins within MS media used. IAA was more effective in the induction of pigmented callus. The pigmented callus cultured in modified MS media containing 2, 4-D showed lower content of shikonin derivatives than those cultured in media including IAA. 

Optimal concentration of hormone is important for maximization of final metabolite concentration in the culture. The optimal amount of hormone (0.2 mg.L^-1^ IAA) was determined based on its effect on productivity of the pigment and applied in the subsequent works. In addition, shikonin synthesis of nonpigmented calli treated with various concentrations of 2, 4-D in MS medium induced when they transferred to the MS/2 medium supplied 0.2 mg.L^-1^ IAA.

*Fresh weight calculations*

Callus growth rate was associated with hormone type under the same condition of medium. It is validated that CIRFW frequency in MS medium significantly is higher than other media. Figure 3 displays that the highest fresh weight was obtained when the calli were cultured in a liquid medium containing 2,4-D whereas the lowest rates of cell division observed when the culture medium included IAA in spite of its high ability for production of red pigment. As revealed in Figure 4 there is also a significant difference between 2,4-D, IAA and free hormone medium. It was found that the 2, 4-D hormone had noticeable influence on callus growth. Several researches proved that callus growth depends on the kind of medium (14, 23). This project reveals that both hormones and culture medium types can be impressive.

*Polyphenol measurment*

Polyphenolic compounds are benefit constituents of medicinal plants due to their strong antioxidant activity. This activity is mostly performed by preventing hydroperoxide conversion into reactive oxyradicals, chelating redox-active metal ions and inactivating lipid free radical chains. The relationship between polyphenol compounds yield and varied hormones and 14 day and 28 day of cultured calli on MS liquid medium in *O. bolbutrichum* cultures are presented in Figure 5. Spectrophotometric analysis of calli with medium extracts revealed that the most significant enhancement of polyphenol production in MS media in combination with 0.2 mg.L^-1^ IAA was observed, with volumetric naphtoquinon yields of 635.6 and 928.7 µg/mg after 2 and 4 weeks, respectively. The yields of total naphtoquinons on modified MS media containing 0.2 mg.L^-1^ 2, 4-D after 2 and 4 weeks were 446 and 463 µg.mg^-1^ were respectively. Reaching at the stationary phase of growth, total content of pigments is enhanced in parallel with it from 2 weeks until 4 weeks (24). 

*HPLC analysis*

Linear standard curve was plotted by five replicates with each standard. High correlation coefficient and good linear calibration curve was obtained for the reference compound (R^2 ^> 0.994). After a 4-weeks culture period, the extract of callus cultures growing in MS/2 liquid medium supplemented with 0.2 mg.L^-2^ IAA along with its medium were analyzed for phytochemical characterization. Shikonin was identified and quantified in the callus and its medium extracted as well as in the roots of the natural plant (Figure 6). Shikonin content in sample and intact root (control) are presented in Table 2. Calli and its medium after 4 weeks of growth show a shikonin production about 2.6 times higher than in the root of the wild plant as the bark root where shikonin accumulates in natural conditions (Figure 7). Optimum concentrations and combinations of hormones for callus induction (1 mg.L-1 2, 4-D) and pigment production (0.2 mg.L-1 IAA) were obtained.

## Discussion

Wide applications in Medical and Pharmaceutical fields have attracted interests in producing natural sources of shikonin/alkanin derivatives (25, 26). Tissue culture is the most appropriate method for accurate usage of these components that can be resulted in preservation of this endangered species. There are no previous reports of seed germination and callus induction of *O. bolbutrichum*. In this research, a simple *in-vitro* technique for rapid germination (after 7 days) and callus initiation (after 10 days) was exerted. Previous researches on initiation or production of shikonin derivatives in the cell suspension cultures of other members of Boraginaceae have proven that both cell growth rate and production of shikonin depend on the kind of the media, specially the source of nitrogen in media (27). Likewise, constant production of these metabolites was performed in the culture media when nitrate served as nitrogen source. It has been proven that the synthesis of shikonin derivatives would not happen when ammonium ion is exerted as the source of nitrogen (28). Therefore, it was decided to remove ammonium and replace it by nitrate. Mizukami *et al.* have reported that both growth and formation of shikonin derivatives were increased in presence of 5% w/v of sucrose concentration (5). Thus, 5% w/v concentration of sucrose for germination as well as induction and maintenance of callus was selected. The effect of hormone types on callus induction and naphthoquinone production has been previously reported in other Boraginaceous family plants (16, 20, 27, 29 and 30). Outcome of this research also represented the significance of the auxins on the callus growth and red pigment production. The effects of kinetin towards different auxins on *Onosma *cultures are shown in Figures 1 and 2. Results of the present study, similar to above mentioned researches, confirmed that pigment formation in *in-vitro* culture of Boraginaceae family plants was inhibited by 2, 4-D. It probably can be a reason for lowest percentage of pigmented callus treated with 2, 4-D. Several tissue culture methods have been carried out for production of naphtoquinones at large scale in many members of Boraginaceous species. In this research, it has suggested that shikonin synthesis is stimulated by the IAA phytohormone (16, 26 and 27). Likewise, as last works, the pigment content increased when callus of *O. bolbutrichum *was grown on culture medium containing IAA hormone and the highest percentage pigment production of callus tissues was belonged to 0.2 mg.L^-1^ IAA. The extensive investigation of the production of shikonin and its derivatives through the callus tissue indicated that shikonin acetate was major constitute within other plants of this family. Based on these findings, as shown in Figure 6, likely a long pick emerged after shikonin may be related to shikonin acetate. 

## Conclusion

To sum up, the outcomes reveal that callus culture is a suitable method for production of valuable natural secondary metabolites biosynthesized by scarce and endangered plant species. In this study, some changes in culture conditions such as culturing and maintaining media as well as applying different concentration of two type auxin hormones increased the content of shikonin and its derivatives as active pharmaceutical ingredients. The results showed that *O. bolbutrichum *can be introduced as a considerable source for scale up production of hydroxynaphtoquinones.
